# Long-term impact of digital media on brain development in children

**DOI:** 10.1038/s41598-024-63566-y

**Published:** 2024-06-06

**Authors:** Samson Nivins, Bruno Sauce, Magnus Liebherr, Nicholas Judd, Torkel Klingberg

**Affiliations:** 1 Department of Neuroscience, Karolinska Institutet, Stockholm, Sweden; 2 Department of Biological Psychology, Vrije Universiteit Amsterdam, Amsterdam, The Netherlands; 3 Department of General Psychology: Cognition, University Duisburg-Essen, Duisburg, Germany

**Keywords:** Videogames, Social media, MRI, Brain, Children, Polygenic scores, Brain imaging, Magnetic resonance imaging, Paediatric research

## Abstract

Digital media (DM) takes an increasingly large part of children’s time, yet the long-term effect on brain development remains unclear. We investigated how individual effects of DM use (i.e., using social media, playing video games, or watching television/videos) on the development of the cortex (i.e., global cortical surface area), striatum, and cerebellum in children over 4 years, accounting for both socioeconomic status and genetic predisposition. We used a prospective, multicentre, longitudinal cohort of children from the Adolescent Brain and Cognitive Development Study, aged 9.9 years when entering the study, and who were followed for 4 years. Annually, children reported their DM usage through the Youth Screen Time Survey and underwent brain magnetic resonance imaging scans every 2 years. Quadratic-mixed effect modelling was used to investigate the relationship between individual DM usage and brain development. We found that individual DM usage did not alter the development of cortex or striatum volumes. However, high social media usage was associated with a statistically significant change in the developmental trajectory of cerebellum volumes, and the accumulated effect of high-vs-low social media users on cerebellum volumes over 4 years was only β = − 0.03, which was considered insignificant. Nevertheless, the developmental trend for heavy social media users was accelerated at later time points. This calls for further studies and longer follow-ups on the impact of social media on brain development.

## Introduction

Children are increasingly engaged with digital media (DM) more than ever before. For example, in the U.S., children aged 8–12 years, on average, spend 4 h and 44 min daily on DM for entertainment purposes^1^, in addition to its use during school and homework. This rise in usage has sparked concerns among parents, caregivers, and policymakers regarding its potential adverse effects on the developing brains of children. However, research in this domain remains inconclusive and somewhat contradictory.

Concerning the DM's impact on cognitive outcomes, prior studies have reported both beneficial and detrimental associations^2–5^. Similarly, a recent review on brain development simply noted that DM's effects can be both positive and negative^6^. This inconsistency in findings can be attributed to several factors. First, the general term ‘digital media’ encompasses a wide range of activities, each potentially influencing development in distinct ways or even exerting contrasting effects. Therefore, it is crucial to differentiate between various digital activities, such as playing video games, watching television/videos, and using social media. Second, the age of the participants is a significant factor. For example, research by Orben et al. in 2022 showed that social media use could negatively affect psychological well-being during particular developmental stages, with these stages occurring at different times for boys and girls^7^. In another study, Soares et al. found that boys who spent more time watching television or playing video games at 11 years old, and more time using computers at 11 and 15 years old, showed improved working memory performance at 22 years old^8^. However, this association was not observed in girls. Third, and perhaps most critical, is the conflation of evidence from cross-sectional and longitudinal studies in reviews. Cross-sectional studies can identify correlations but cannot establish causality. Whereas longitudinal studies may even yield opposite results. For example, a longitudinal study using structural equation modeling has found a negative correlation between time spent playing video games and intelligence^4^. However, when controlling for baseline cognition and other background variables, the longitudinal analysis revealed that playing video games positively influenced changes in intelligence (β = 0.17). The initial negative correlation between video gaming and cognitive performance was interpreted as resulting from self-selection.

Longitudinal research on the effect of DM and brain development in children remains limited. A series of studies on a cohort of Japanese children observed that watching television increased grey matter volume in frontal areas^9^, playing video games increased mean diffusivity in the white matter^10^, and internet usage decreased grey matter volume in extensive brain regions^11^. Although informative, this is a single cohort of less than 300 individuals, which varied widely in age, between ages 6 to 18. Brain development during this period is nonlinear, which was not accounted for in the statistical modeling. In 2023, Miller et al. assessed the impact of DM on functional connectivity over 2 years in a cohort of over 4000 children^12^. They reported no effects exceeding a size of 0.2, the predetermined threshold for significance.

The ongoing debate over what constitutes a meaningful effect size continues without consensus in psychology and neuroscience. This issue is particularly relevant in large-scale studies like the Adolescent Brain Cognitive Development (ABCD) study, where statistical significance may not equate to a meaningful effect for the individual^13^. The traditional criteria by Cohen, which categorizes effect sizes of 0.2 as small and 0.5 as medium, were arbitrary from the outset, with Cohen himself acknowledging the lack of solid evidence for these benchmarks^14^. Funder and Ozer propose that effect sizes must be contextualized, and propose as guidelines that an r-value of 0.05 indicates a very small effect and 0.1 a small effect. The frequency of an event may also be crucial, as repeated events can accumulate effects over time, according to Abelson^15^.

Furthermore, when interpreting effect sizes, it is essential to consider additional factors. Even a small effect can have significant implications if it influences various aspects of an individual's life or interacts positively with other variables. Habituation or counteractive responses might mitigate an effect's impact^16^. In our analysis, we regard an annual effect size of 0.05 as meaningful. This threshold is deemed appropriate, considering the cumulative influence of DM and the potential for an effect on a general ability like attention to significantly impact schooling and everyday life.

Our study aimed to investigate the individual effects of DM usage on structural brain development in children aged 9.9 years at baseline (T_0_) over 4 years, adjusted for age, sex, SES, scanner sites, and genetic predisposition. We selected global cortical surface area (CSA) as the main outcome measure since previous studies have shown a strong relationship between global CSA and intelligence across different age groups^17–22^. Moreover, we used cortical surface area rather than cortical thickness because studies have consistently reported an association of environmental variables, such as SES, on cortical surface area rather than on cortical thickness^23^.

We further investigated the individual brain structures, i.e., the volumes of the striatum and cerebellum, which have been implicated in cross-sectional studies of DM usage^24,25^. In general, global CSA tends to increase during this period of childhood a part of normal development with a peak age at 11 years of age^26^. Building upon our prior research findings^4^, we hypothesized that DM usage, particularly playing video games, would be associated with an increase in global CSA. Since DM usage differs between sexes^27^, we will study the effect of sex on these relationships.

## Results

### Baseline characteristics

Of the 11,875 children from the ABCD study cohort, 6469 children (age, mean (SD) = 9.9 (0.6) years; boys, n (%) = 3369 (52.1%)) fulfilled our inclusion criteria and were included at the T_0_ visit (i.e., 9–11 years of age). Of these, 4610 children (age = 12.0 (0.7) years; boys = 2487 (53.9%)) were included at the T_2_ (i.e., 2 years later) visit, and 1697 children (age = 13.4 (0.6) years; boys = 949 (55.9%)) were included at the T_4_ (i.e., 4 years later) visit. 1462 children had usable data for all three-time points.

The estimated time spent by these children on DM types at T_0_ was 0.5 h/day for using social media, 0.9 h/day for playing video games, and 2.1 h/day for watching television/videos (Table 1).

**Table 1:** Descriptive characteristics of the sample.

Variables	Overall	Boys	Girls
N at T_0_	6469	3369	3100
Age, years at T_0_	9.9 (0.6)	9.9 (0.6)	9.9 (0.6)
Socioeconomic status	0.01 (0.99)	0.03 (0.94)	-0.02 (1.00)
Polygenic score	0.02 (1.00)	0.04 (0.98)	-0.003 (1.02)
Digital media usage			
Using social media			
T_0_	0.49 (1.06)^a^	0.39 (0.95)^a^	0.56 (1.09)^a^
T_1_	0.84 (1.49)^b^	0.66 (1.31)^b^	0.99 (1.59)^b^
T_2_	1.22 (1.33)^c^	0.89 (1.16)^c^	1.47 (1.38)^c^
T_3_	1.87 (1.45)^d^	1.47 (1.35)^d^	2.22 (1.45)^d^
T_4_	2.50 (1.45)	2.19 (1.49)	2.76 (1.36)
Watching television/videos			
T_0_	2.13 (1.69)^a^	2.20 (1.72)^a^	2.05 (1.65)^a^
T_1_	2.36 (1.77)^b^	2.44 (1.80)^b^	2.26 (1.73)^b^
T_2_	2.21 (1.24)	2.24 (1.24)	2.13 (1.23)
Playing video games			
T_0_	0.95 (1.06)^a^	1.24 (1.15)^a^	0.64 (0.84)^a^
T_1_	1.15 (1.18)^b^	1.50 (1.23)^b^	0.78 (0.99)^b^
T_2_	1.48 (1.39)^c^	1.94 (1.37)^c^	0.93 (1.19)^c^
T_3_	1.81 (1.47)^d^	2.33 (1.36)^d^	1.21 (1.35)^d^
T_4_	2.00 (1.57)	2.53 (1.40)	1.37 (1.51)
Average estimated time spent over four years on			
Using social media	1.35 (0.95)	1.12 (0.87)	1.60 (0.99)
Watching television/videos	2.24 (1.27)	2.30 (1.28)	2.16 (1.25)
Playing video games	1.47 (1.02)	1.91 (0.96)	0.98 (0.86)
Playing mature video games at T_0_	0.53 (0.84)	0.76 (0.94)	0.27 (0.60)
Watching mature movies at T_0_	0.34 (0.60)	0.38 (0.62)	0.30 (0.58)
Total screen time—reported by children at			
T_0_	3.60 (2.93)	3.86 (2.99)	3.27 (2.76)
T_1_	4.32 (3.38)	4.58 (3.33)	4.03 (3.42)
Total screen time—reported by parents at			
T_0_	2.90 (2.32)	3.04 (2.37)	2.73 (2.23)
T_1_	3.17 (2.62)	3.28 (2.58)	3.05 (2.65)

Data are presented as the mean (standard deviation).

T_0_, baseline visit; T_1_, first-year visit; T_2_, second-year visit, T_3_, third-year visit; and T_4_, fourth-year visit. A two-sample t-test was carried out to determine the differences between the visits.

^a^Significant differences between T_0_ and T_1_.

^b^Significant differences between T_1_ and T_2_.

^c^Significant differences between parent and child reported at T_0_.

^d^Significant differences between parent and child reported at T_1_.

^e^Significant differences between parent and child reported at T_2_. The average estimated time spent over 2 years is calculated by the average across all three annual visits. The total screen time reported by children is calculated by summing the estimated time spent on individual digital media types. For example, total screen time at T_0_ is calculated by summing the estimated time spent using social media, watching television and videos, and playing video games at T_0_.

Compared to the T_0_ visit, the estimated time spent using DM types significantly increased over 4 years in the overall cohort and in boys and girls (Table 1). During the 4 years of the follow-up period (i.e., across all four annual visits), children, on average, spent 1.4 h/day using social media, 1.5 h/day playing video games, and 2.2 h/day watching television/videos. Moreover, during this period, boys spent more time playing video games or watching television/videos, whereas girls spent more time using social media or watching television/videos.

As expected, parents reported less total screen time use per day in children compared to child reports across two visits (Table 1).

### Normal brain developmental trajectory

**Figure 1:**
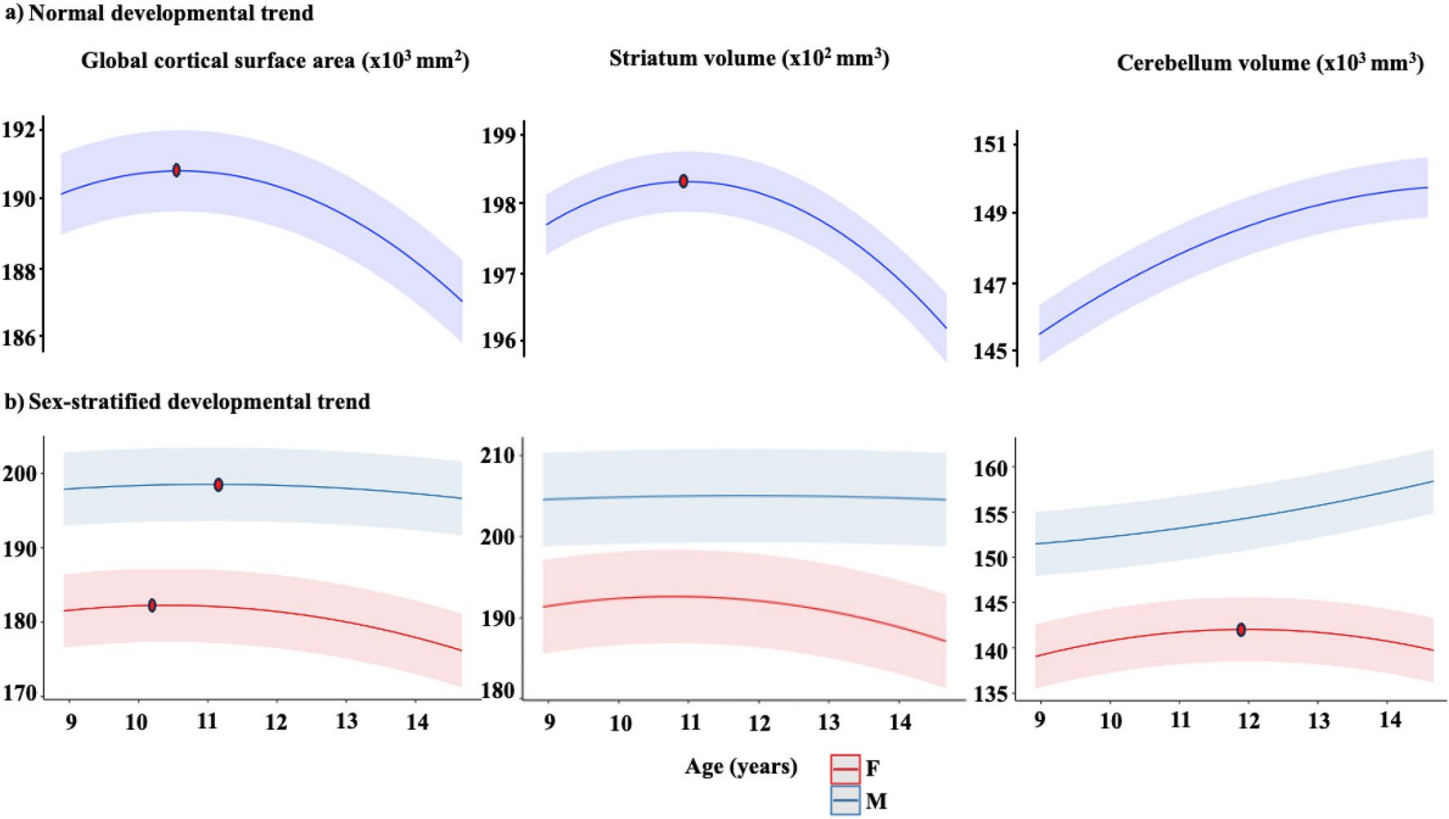
(**a**) Plots representing the quadratic effects of age (years) predicting global cortical surface area, striatum and cerebellum volumes in the overall cohort, adjusted for sex, socioeconomic status, polygenic scores for cognitive performance, and 20 principal components, and the grey shade around the regression lines corresponds to a 95% confidence interval of the intercept; (**b**) sex-stratified developmental trend adjusted for the same covariates as mentioned above. The dots represent the peak age, estimated by the first derivative. The y-axis represents brain structures.

Overall development followed an inverted U-shaped developmental trend for global CSA, striatum and cerebellum volumes between mid-childhood and early adolescence, i.e., age-related increase during mid-childhood and subsequently decrease during early adolescence. According to the fitted model, the global CSA, striatum, and cerebellum volumes peaked at 10.6, 10.9, and 15.4 years, respectively (Fig. 1a).

### Sex effect on brain development

To determine whether the trajectory differed between the sexes, we added interaction terms (age * sex; age^2^ * sex) to the pre-existing model. There was a significant interaction for sex with global CSA and cerebellum volumes, but not for striatum (eTable 2).

Overall, boys had larger global CSA and cerebellum volumes than girls (eTable 3; Fig. 1b), but the peak was much earlier in girls (global CSA = 10.4 years and cerebellum = 11.9 years) than in boys (global CSA = 11.1 years). Cerebellum volumes increased over this age range for boys.

### SES and cogPGS

**Figure 2:**
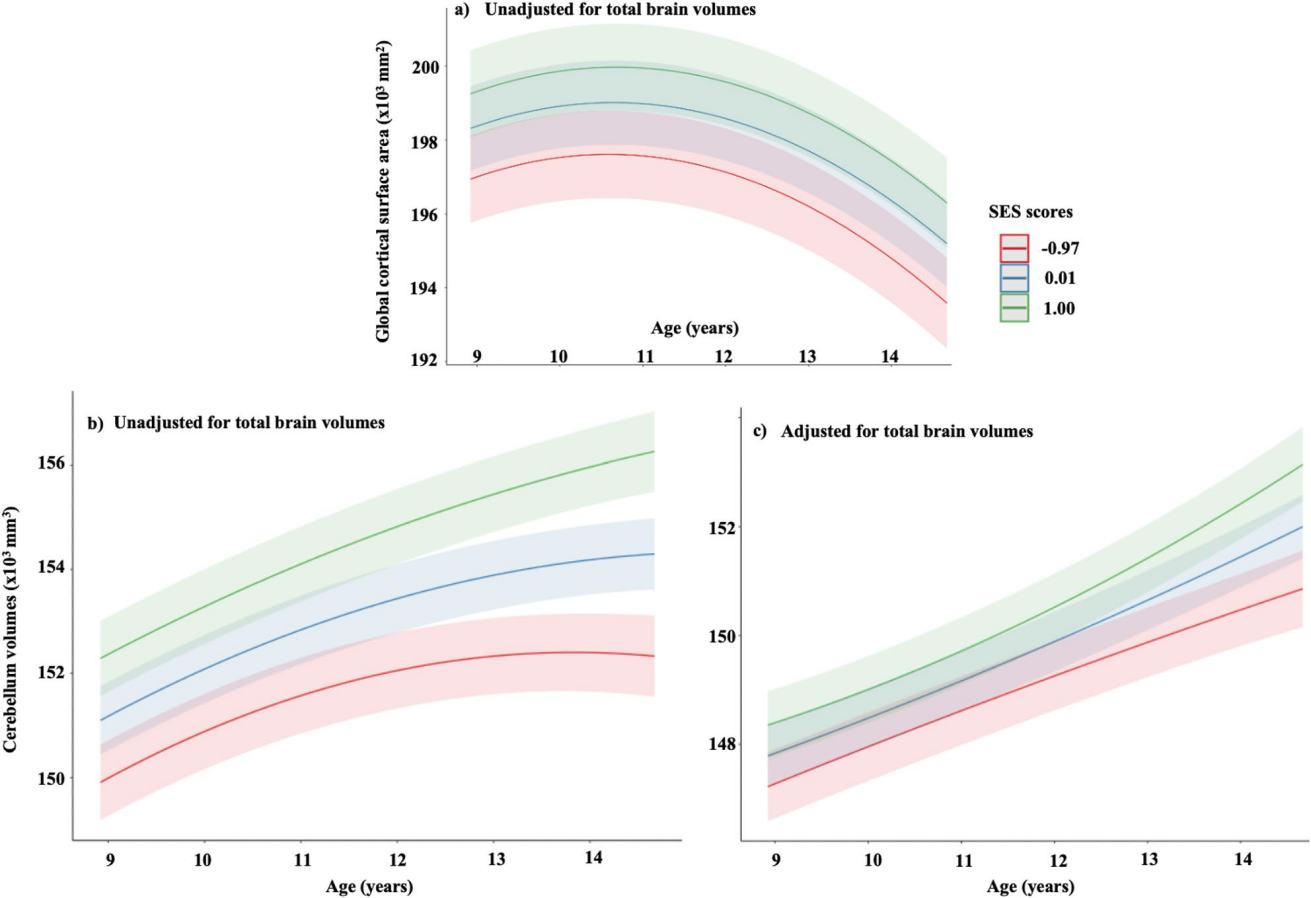
Developmental trajectories and socioeconomic status, (**a**) global cortical surface area, and (**b**,**c**) cerebellum (presented as adjusted and unadjusted for total brain volumes (TBV)). For visual purposes, we present in age (years). socioeconomic status (SES) is categorized into quartiles using ggpredict [quart2] function in R.^89^ Children from low levels of socioeconomic status had a relatively smaller global cortical surface area or cerebellum volumes and accelerated maturation of the brain compared to their developing peers. *SES* socioeconomic status.

**Figure 3:**
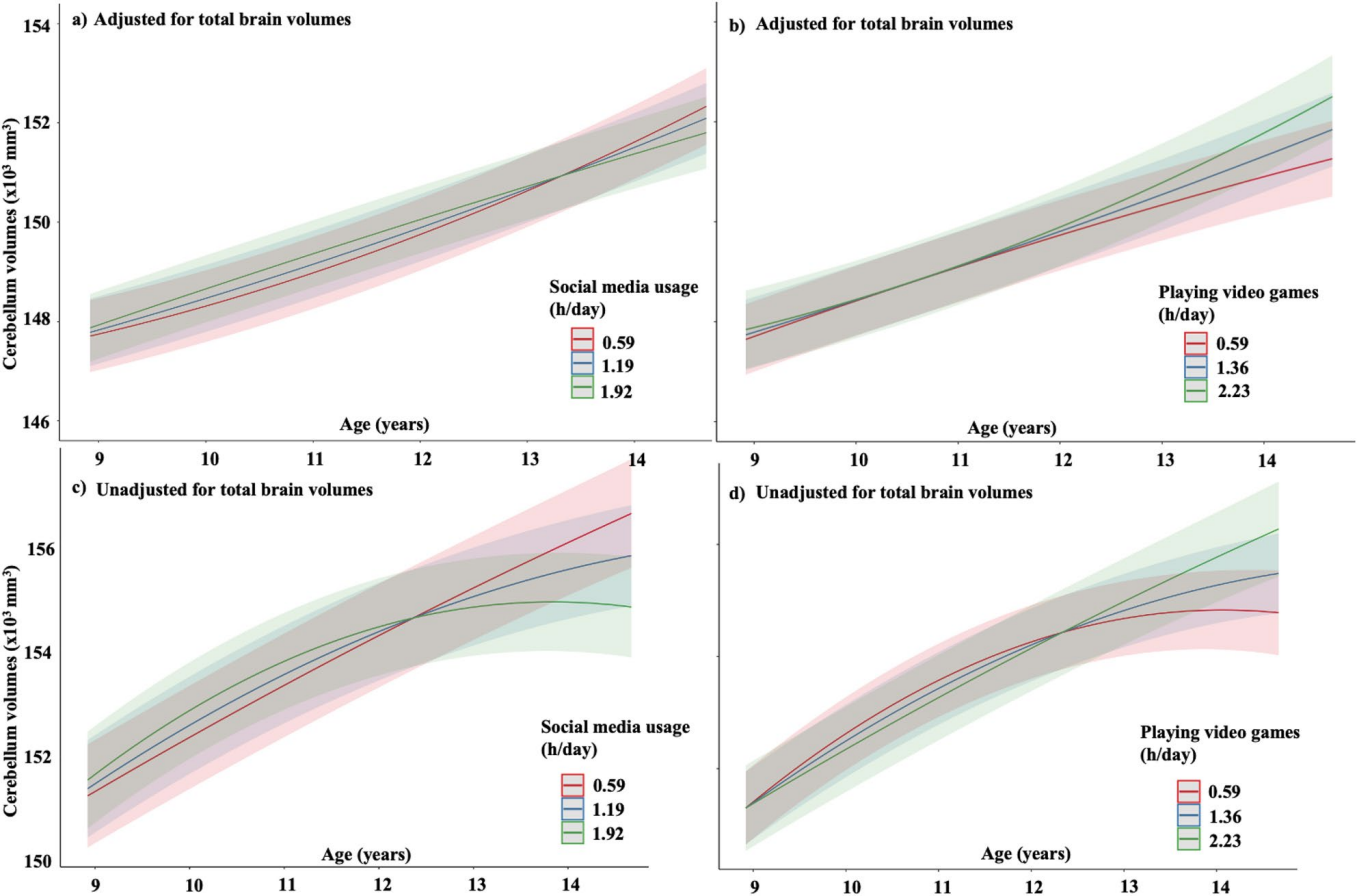
Relationship between digital media usage and cerebellum development over time. The interactions presented in **(a,c)** social media usage **(b,d)** playing video games, and time^2^ on the cerebellum development; however, they are presented in age (years) for visual purposes. Digital media usage is categorized based on quartiles using ggpredict [quart2] function in R.^89^ Children who spent a longer time on social media usage **(a,c)** had a decrease in cerebellum volume. Similar findings were seen for those who spent on mean levels. In contrast, children who spent a longer time playing video games **(b,d)** had an increase in cerebellum volume.

Overall, we observed a positive association between SES and global CSA (β = 0.08 to 0.11; p < 0.001) (Table 2). For illustration purposes, we additionally categorized SES into quartiles and plotted global CSA development across them (Fig. 2). Similarly, we categorized SES into three quartiles and studied the trend for cerebellum volumes (Table 2). Overall, children from lower levels of SES had lower global CSA and cerebellum volumes compared with their developing peers and had earlier maturation (Fig. 3).

**Table 2:** Association between individual digital media exposure and brain outcomes in children aged 9–11 years with 4 years of follow-ups.

Brain regions	Social media usage		Time (linear)		Time (Quadrant)		SES		cogPGS		Social media usage x Time		Social media usage x Time^2^	
	β (SE)	p	β (SE)	P	β (SE)	p	β (SE)	P	β (SE)	P	β (SE)	P	β (SE)	P
Global cortical surface area (mm^2^)	0.001 (0.001)	0.67	0.04 (0.001)	< 0.001	− 0.03 (0.01)	< 0.001	0.11 (0.02)	** < 0.001**	0.05 (0.02)	0.005	0.001 (0.006)	0.36	− 0.01 (0.004)	0.004
Striatum Volume (mm^3^)	− 0.007 (0.01)	0.49	− 0.02 (0.01)	0.11	− 0.004 (0.005)	0.42	0.01 (0.02)	0.43	− 0.01 (0.02)	0.40	− 0.007 (0.006)	0.91	0.001 (0.003)	0.96
Cerebellum Volume (mm^3^)	0.02 (0.01)	0.10	0.09 (0.008)	< 0.001	0.01 (0.005)	0.04	0.05 (0.02)	0.004	− 0.002 (0.02)	0.98	0.02 (0.005)	** < 0.001**	− 0.02 (0.003)	** < 0.001**

	Playing video games		Time (linear)		Time (Quadrant)		SES		cogPGS		Playing video games x Time		Playing video games x Time^2^	
	β (SE)	p	β (SE)	P	β (SE)	p	β (SE)	P	β (SE)	P	β (SE)	P		
Global cortical surface area (mm^2^)	− 0.04 (0.01)	< 0.001	0.03 (0.01)	0.001	− 0.05 (0.01)	< 0.001	0.08 (0.02)	** < 0.001**	0.03 (0.02)	0.16	− < 0.001 (0.005)	0.95	0.008 (0.003)	0.01
Striatum Volume (mm^3^)	− 0.01 (0.01)	0.16	− 0.001 (0.01)	0.71	− 0.01 (0.01)	0.10	0.02 (0.02)	0.20	− 0.009 (0.02)	0.59	− 0.01 (0.006)	0.07	0.004 (0.003)	0.16
Cerebellum Volume (mm^3^)	0.006 (0.009)	0.49	0.12 (0.008)	< 0.001	− 0.02 (0.005)	< 0.001	0.06 (0.02)	**0.002**	− 0.006 (0.02)	0.69	− 0.008 (0.005)	0.07	0.01 (0.003)	** < 0.001**

	Watching television		Time (linear)		Time (Quadrant)		SES		cogPGS		Watching television x Time		Watching television x Time^2^	
	β (SE)	p	β (SE)	p	β (SE)	p	β (SE)	P	β (SE)	P	β (SE)	P	β (SE)	P
Global cortical surface area (mm^2^)	− 0.03 (0.008)	< 0.001	0.04 (0.01)	0.001	− 0.04 (0.006)	< 0.001	0.08 (0.02)	** < 0.001**	0.04 (0.02)	0.05	− 0.003 (0.004)	0.50	− 0.001 (0.002)	0.63
Striatum Volume (mm^3^)	− 0.02 (0.001)	0.03	− 0.03 (0.01)	0.03	0.003 (0.007)	0.62	0.02 (0.02)	0.28	− 0.005 (0.02)	0.78	− 0.003 (0.005)	0.54	− 0.002 (0.002)	0.30
Cerebellum Volume (mm^3^)	0.007 (0.007)	0.33	0.08 (0.009)	< 0.001	0.006 (0.006)	0.34	0.03 (0.02)	0.11	0.03 (0.02)	0.10	0.01 (0.003)	0.007	− 0.006 (0.002)	0.02

P values presented are uncorrected for multiple comparisons. The main effects that survived a number of tests were highlighted (p < 0.05/18 = 0.0027). Abbreviations: SES, socioeconomic status; cogPGS, polygenic scores for cognitive performance.

Model :$${Y}_{ij}={\beta }_{0}+{\beta }_{1}{cogPGS}_{i}+{\beta }_{2}Poly({Time, 2)}_{ij}+{\beta }_{3}{Sex}_{i}+{\beta }_{4}{Age at baseline}_{i}+{\beta }_{5}{DM Usage}_{i}+ {\beta }_{6}{SES}_{i}+ {\beta }_{7}{Ancestry}_{i}+ {\beta }_{8}\left({DM Usage}_{i}*Poly({Time, 2)}_{ij}\right)+{\beta }_{9}({DM Usage}_{i}*Poly({Time, 2)}_{ij}*{SES}_{i}) +{{\beta }_{10}\left({DM Usage}_{i}*Poly\left({Time, 2)}_{ij}*{cogPGS}_{i}\right)+\left(1 \right|sites\right)+ \upsilon }_{i0}+{ \upsilon }_{i1}Poly({Time, 2)}_{ij}+{\varepsilon }_{ij}$$

No association was found between cogPGS and global CSA and the volumes of cerebellum and striatum.

There was no significant three-way interaction found between DM usage, time, and SES on any brain regions studied (i.e., global CSA, and volumes of striatum and cerebellum).

### Interaction of DM usage and time on brain development

There were multiple interactions between the average DM usage and both linear and quadratic effects of time on brain development (Table 2). Only the significant interactions that survived Bonferroni corrections (p < 0.003) will be discussed below.

### Social media usage

We found a significant interaction between average social media usage and both linear and quadratic effects of time (i.e., average social media usage x time and average social media usage x time^2^, respectively) with cerebellum volume. Here, we observed a positive association of social media usage and time with cerebellum volumes for a linear term (β = 0.02) but a negative association of social media usage and time with cerebellum volumes for a quadratic term (β = − 0.02) (Table 2). The consequence of these effects is illustrated in (Fig. 3a,c); there is a slight difference in trajectory, which results in an earlier decline and lower volume at the last time-point.

There was no significant interaction between social media usage and time with other brain regions studied (i.e., global CSA and striatum volumes) (Table 2).

### Playing video games

In contrast to social media usage on brain development, we observed a significant positive interaction between average time spent playing video games and a quadratic effect of time (but not linear) with cerebellum volume (β = 0.01) (Table 2). This resulted in a trajectory with continued increase throughout the study period and a larger cerebellar volume at the last time-point (Fig. 3b,d).

There was no significant interaction between playing video games and time in other brain regions studied (i.e., global CSA and striatum volumes) (Table 2).

### Watching television/videos

There was no significant interaction between watching television and time with any of the brain regions studied (i.e., global CSA, volumes of cerebellum and striatum) (Table 2).

There were no significant three-way interactions between any DM usage, time, and sex with the brain structures studied. Therefore, we did not carry out separate analyses for boys and girls (eTable 4).

### Additional analysis

In investigating whether DM estimates preceded changes in cerebellum volume, we observed a negative trend for high social media users with changes in cerebellum volumes (β = − 0.01; p = 0.10). Conversely, there was a significant positive association for high video game users with changes in cerebellum volumes (β = 0.02; p =  < 0.001) (eTable 5).

We then investigated whether social media usage at T_0_ could predict total changes in cerebellum volumes (T_4_–T_0_) and found that social media usage at T_0_ was not associated with changes in cerebellum volumes (β = − 0.03; p = 0.16) (eTable 6). The estimate (β = − 0.03) thus represents the overall effect size of social media usage over a 4-year study period.

Furthermore, after excluding time spent on video chatting or texting from social media usage, we still found that the direction of the observed effects between average social media usage on cerebellum volumes remained significant, with the same effect size (β = − 0.02; p = 0.002).

### Sensitivity analyses

When we excluded children who were born preterm, had low birth weight (< 2500 g), or had ADHD diagnosis, 3979 children fulfilled the criteria for eligibility, and the findings of the average social media usage or playing video games and cerebellum volumes remained significant (eTable 7).

Further, our analysis was confined to children with MRI data available across all three visits (n = 1462), and despite this restriction, the observed effects between average social media usage or playing video games and cerebellum volumes remained significant (eTable 8).

## Discussion

In this large prospective cohort study, we studied the long-term effects of DM usage on the development of the cortex, striatum, and cerebellum across three time points spanning between mid-childhood to adolescence, in children aged 9 to 11 years. Despite our initial hypothesis, we found that DM usage did not significantly alter the development of the global CSA or striatum volume. However, children who devote more time to playing video games had a weak increase in cerebellum volume during the critical developmental window of development (β = 0.01), while those who spent more time using social media had a subtle decrease in cerebellum volume (β = − 0.02). These associations persisted in subsequent analysis, even when factors such as preterm birth, lower birth weight, or those with ADHD diagnosis, were excluded, underscoring the robustness of our findings. And these associations also did not differ between the sexes. However, the effect size observed for this association was smaller than our predefined threshold of 0.05. Moreover, in analysing the accumulated differences in cerebellum volumes over 4 years were also very small, which is likely not of relevance to the individual. Nevertheless, this difference was accelerated during the last year (Fig. 2). Thus, it is relevant to conduct further research to analyse the long-term effects of social media on brain development.

The term “social media” consists of a broad spectrum of digital tools associated with social interaction, including social networking sites, text messaging applications, and video chatting. Previous studies examining the association between social media use and functional or neural outcomes in both children and adolescents have often either combined all these digital tools under the umbrella term “social media use”^4,28^, or scrutinized them separately, distinguishing between social media platforms (e.g., Facebook) and social communication tools (e.g., text messaging) in their analyses^29,30^. Consistent with previous studies we first investigated the effect of social media usage on brain development by combining all these digital tools. We specifically included activities related to social media platforms and studied their singular long-term effect on cerebellum development. Even in this refined analysis, we still observed a persistent weak negative effect of social media usage on cerebellum volumes.

If the negative developmental trend for the cerebellum persists, it might be of significant concern, particularly considering that adolescence serves as the period when many psychiatric disorders have their onset^31,32^. Moreover, consistent findings report an association between cerebellum abnormalities with various psychiatric disorders, such as depression and anxiety disorders^33^. In addition, the cerebellum is a core component of the neural circuitry underpinning many cognitive deficits associated with ADHD, including working memory, response inhibition, attention shifting, and processing of rewards and temporal information^34–37^.

The cerebellum is sensitive to environmental exposures both prenatally, as demonstrated by studies of maternal alcohol, maternal diabetes, hypoxia, and postnatal glucocorticoid exposure^38,39^, and postnatally^40^. In our study, we observed that children from lower SES quartiles had smaller cerebellum volumes, providing further for the susceptibility of the cerebellum structure to environmental factors^41,42^. The transition from childhood to adolescence represents a critical developmental phase characterized by hormonal and physiological changes, including myelination, strengthening of synapses, and selective pruning of neurons and connections. Social media users often contend with constant distractions, which can significantly impact their behavior, leading to inattention symptoms^43^. Additionally, these users can become easily diverted from tasks like reading or homework, etc. Moreover, the use of social media necessitates continual response to stimuli, decision-making, and the execution of motor movements, among various other cognitive and behavioral tasks. Previous studies on social media usage have consistently reported negative effects on life satisfaction^7^, overall well-being^44^, and depressive symptoms^45^, among adolescents. Based on these observations, one might speculate that a distinct window of susceptibility to emotion and frequent shifts in task stimuli might be key contributing factors to the observed decrease in cerebellum volumes. At the neuronal level, this could reflect the acceleration of the natural process of synaptic pruning and changes in myelination among high social media users, which would then appear as a decrease in cerebellum volume at a later time point.

Consistent with prior research^46,47^, we observed an inverted U-shaped trajectory in the development of the cortex during mid-childhood and adolescence, with girls reaching their peak earlier than boys. These findings align with histological studies suggesting continued myelination and reduction in synaptic density during adolescence^48,49^. At a microscopic level, cortical maturation involves synaptic overproduction in childhood, followed by selective elimination and strengthening of connections later in development^50^. During these stages of development, environmental exposure might guide selective synapse elimination in adolescence^51,52^. Supporting this notion, we found that children from lower SES quartiles exhibited smaller global CSA across development compared to their peers.

Although this is a longitudinal study with a large number of participants, the study has some notable limitations. First, this is an observational study, and therefore, we cannot establish causal inference. However, we adjusted for most of the covariates such as age, sex, SES, and genetics. Additionally, to mitigate selection bias, we ensured the inclusion of only one child per family. Second, the estimated time spent on various DM types was self-reported, introducing potential recall or accuracy bias. Nevertheless, it should be noted that studies have reported high test and retest reliability of self-reported behaviors among adolescents^53^. Third, the survey questionnaire utilized to capture DM usage from T_2_ visits onwards was modified compared with T_0_ or T_1_ visits in response to technological advancements and the heightened usage of DM among adolescents. However, we harmonized the survey questionnaires from the T_2_ visit onwards to maintain consistency with the earlier visits. Fourth, the response measure for the survey questionnaire in both T_0_ and T_1_ visits was set between ‘0 and 4+ hours’; this is one of the major drawbacks of the ABCD questionnaire. For example, a child who spent four hours engaged in video games or using social media would receive the same score as a child who spent 12 h, despite the significant difference in their exposure. Fifth, the ABCD questionnaire failed to capture information regarding the timing of DM usage, either during the day or night, thus impeding the exploration of the potential effects of bedtime DM usage on brain development. Finally, the survey questionnaires used in this study failed to capture any information regarding the genre of video games. Given that different activities and actions of video gaming may exert distinct impacts on brain development.

In summary, DM usage, particularly playing video games, does not alter cortical brain development during the 4-year window, but social media usage is weakly associated with a decrease in cerebellum volumes, a trend that was accelerated at later time-points. These findings should be continued by longer follow-up, and more detailed documentation of DM usage, but is a cause for concern regarding the usage of social media in children and adolescents.

## Methods

### Participants

The neuroimaging and behavioral data used in this study were obtained from the ABCD Study (data release 5.0; https://abcdstudy.org/; 10.15154/1523041), a longitudinal cohort of 11,875 children born between 2005 and 2009. These children were enrolled at ages 9–11 years from 21 research sites across the U.S. between 2016 and 2018^54^, with the intention of following them for a period of at least 10 years. This recruitment cohort closely matches the sociodemographic composition of the US population of 9–11-year-old children. Most of the children were enrolled through local elementary and charter schools at each data-collection site. A smaller portion was recruited through community outreach and word-of-mouth referrals outside of the school setting. Twins were identified and recruited from birth registries^55,56^.

During each visit, children accompanied by a parent/guardian, completed a series of measures. These included neurocognitive tests, mental and physical health questionnaires, environmental exposure data collection, providing biological specimens, and participating in brain imaging^54,57–61^. All were asked for an in-person assessment session for self- or parent-report of mentioned behavioral measures and for biological specimen collections once a year, with brain imaging conducted biannually. For this study, we used data collected between September 2016 and January 2022, which included baseline (T_0_), 1-year follow-up (T_1_), 2-year follow-up (T_2_), 3-year follow-up (T_3_), and 4 years follow-up (T_4_)^60,61^. Children were excluded if they were born extremely preterm (< 28 weeks of gestation) or had birth weight (< 1200 g), were not proficient in English, had any neurological problems, had a history of seizures, or had a contraindication to undergo brain MRI scans. All children and their parents/guardians provided informed written consent/assent for participation, and the central Institutional Review Board at the University of California, San Diego approved the study protocols. All the research methods were performed in accordance with the relevant guidelines and regulations.

Children who did not have relevant data on either SES, genetics, DM usage; or neuroimaging were excluded from the present study. Additionally, the ABCD cohort included twins and siblings, therefore we randomly selected one child per family to eliminate this source of bias.

### Neuroimaging

Children underwent brain MRI scans on 3-Tesla scanner platforms (Siemens Prisma, Philips, or General Electric 750) using a standard adult-sized head coil at three different time points over a span of 4 years (i.e., T_0_, 2 years later (T_2_), and 4 years later (T_4_)). A standardized protocol for scanning was used to harmonize the scanning sites and MRI scanners. Three-dimensional T1-weighted images (1-mm isotropic) were acquired using a magnetization-prepared rapid acquisition gradient-echo (MP-RAGE) sequence and processed using FreeSurfer software (version 5.3.0)^62^. All the pre-processed images were quality-checked according to the ABCD protocol, as described earlier^39^, and children with excessive head motion or poor image quality were excluded from the current study. In brief, all the imaging data and FreeSurfer outputs were evaluated by the ABCD Data Analysis, Informatics, and Resource Center (DAIRC) image processing pipeline for real-time motion detection and correction^54,57^. In addition, FreeSurfer output was rated manually by a trained technician for the following errors: motion, homogeneity, white-matter underestimation, pial overestimation, and magnetic susceptibility artifacts; and were rated from 0 to 3 (0 = absent, 1 = mild, 2 = moderate, and 3 = severe). As per the ABCD study recommendation, we excluded children with poor scan quality, did not pass manual quality check, or with any incidental findings.

The Destrieux atlas was used to calculate total brain volumes and global cortical surface area (CSA), while the ASEG atlas was used to segment both striatum and cerebellum volumes^57,62,63^.

### Covariates

#### Socioeconomic status

SES was defined as the first principal component from a probabilistic principal component analysis (PCA), capturing 65% of the variance in total household income, highest parental education, and neighbourhood quality. Children missing more than one of these SES measures were excluded. Household income was determined by the combined annual income of all family members over the past 12 months, categorized as less than $49,999 (1), $50,000–74,999 (2), $75,000–99,999 (3); $100,000–199,999 (4); and greater than $200,000 (5). Parental education was categorized into middle school or less (1), some high school (2), high school graduate (3), some college/associate degree (4), bachelor’s degree (5), master's degree (6), or professional degree (7). The neighbourhood quality was determined using the area deprivation index, calculated from the American Community Survey using the address of the primary residency^64^. The SES composite and each subcomponent were normalized (mean = 0, SD = 1).

### Polygenic score derivation and analyses

#### Genotyping, quality control, and imputation

Saliva samples were collected from all the children during the T_0_ visit and genotyped using Rutgers University Cell and DNA repository using the Smokescreen array consisting of 646,247 genetic variants^65^.

Quality control, imputation, and genetic PCA were performed by the National Bioinformatics Infrastructure Sweden (NBIS). The following pre-processing steps were conducted. Briefly, single nucleotide polymorphisms (SNPs) with call rates < 98% or minor allele frequencies (MAFs) < 1% were excluded before imputation. Individuals with high rates of missingness (> 2%) and absolute autosomal heterozygosity > 0.2 were excluded, resulting in 10,069 children and 430,622 genetic variants. Haplotypes were prephased using SHAPEIT2, and genetic markers were imputed using IMPUTE4 software.

We utilized the 1000 Genomes haplotypes—Phase 3 integrated variant set release in NCBI build 37 (hg19) coordinates as reference populations. This dataset consists of 2504 samples and 5008 haplotypes from Europeans, Africans, East Asians, Southern Asians, and Americans (https://mathgen.stats.ox.ac.uk/impute/1000GP_Phase3.html). We used this imputation since it provides better concordance in diverse human populations^66,67^. After that, genotypes with an INFO score < 0.3 or MAF < 0.001% were excluded, which yielded 40,637,119 SNPs in a total of 10,069 children.

The PCA module, as implemented in RICOPILI^68^, was used to check for outliers and control population structure. SNPs were pruned so that there was little linkage disequilibrium (LD) between SNPs (R2 < 0.2, 200 SNP window: Plink–indep-pairwise 200 100 0.2). LD pruning was repeated until 100 K SNPs were reached. The resulting SNPs were then projected into the PCA^69,70^. We utilized the first 20 principal components (20PCs) from the genetic PCA.

### cogPGS calculation

We created polygenic scores for cognitive performance (cogPGS) in each child using PRSice-2^71^, which involved summing the effect sizes of thousands of SNPs (weighted by the presence of effect alleles in each child). These SNPs were discovered by large genome-wide association studies (GWAS) on educational attainment, mathematical ability, and general cognitive ability^72^. Details regarding the effect sizes and p values of their SNPs can be assessed through the Social Science Genetics Association Consortium (https://www.thessgac.org/data).

We utilized the data provided by the consortium from a multitrait analysis of GWAS^73^, which, in our case, represents a joint polygenic score focused on a GWAS of cognitive performance and complemented by information from a GWAS on educational attainment, a GWAS on the highest-level math class completed, and a GWAS on self-reported math ability. This joint analysis is ideal because pairwise genetic correlations of these traits were high^72^, and these GWAS had hundreds of thousands of individuals. Such a large sample size allows new studies to detect effects in samples of a few hundred individuals with 80% statistical power.

To construct the cogPGS, we performed clumping and pruning to remove nearby SNPs that are correlated with each other. The clumping sliding window was 250 kb, with the linkage disequilibrium clumping set to r^2^ > 0.25. We included the weightings of all SNPs, regardless of their p-value from the GWAS (p = 1.00 threshold), resulting in 5255 SNPs. Finally, we normalized (mean = 0, SD = 1) the cogPGS to fairly compare their effects on different phenotypes. For the present study, we used cogPGS to reflect the genetic predisposition of cognitive performance and included 20 genetic principal components (PCs) to account for the possibility of population stratification within the Add Health European-ancestry subsample in the same model.

### Exposure

#### Digital media usage

The estimated time spent on individual DM usage (i.e., using social media, playing video games, or watching television/videos) was assessed at all annual visits (i.e., T_0_, 1 year later (T_1_), T_2_, 3 years later (T_3_), and T_4_) using the self-reported Youth Screen Time Survey.

#### Self-report survey

At each visit, children reported the number of hours they spent on a typical weekday (i.e., Monday to Friday during the school year and holiday/school breaks) as well as weekend days (i.e., Saturday and Sunday). These hours were categorized by device, media platform, or activity excluding the number of hours spent on school-related work. Specifically, they reported the number of hours dedicated to the following activities:

1. watching television or movies,
2. watching videos (e.g., YouTube),
3. playing video games on a computer, console, phone, or another device (e.g., Xbox, PlayStation, iPad),
4. Texting on a cell phone, tablet, or computer (e.g., Google Chat, WhatsApp),
5. Visiting social networking sites (e.g., Facebook, Twitter, Instagram), and
6. Using video chat (e.g., Skype, FaceTime).

To be consistent with our earlier study^4^, we categorized DM usage as follows: (a) using social media (4 + 5 + 6), (b) playing video games (3), or (c) watching television/videos (1 + 2). The response options included were none—‘0’, < 30 min—‘0.25’, 30 min—‘0.5’, 1 h—‘1’, 2 h—‘2’, 3 h—‘3’, or > 4 h—‘4’.

To calculate the average hours spent per day for individual DM usage, the following formula was used: [(total number of hours spent on a weekday * 5) + (the total number of hours on a weekend day * 2)]/7.

For both the T_0_ and T_1_ visits, data were collected using the same categorical scale as described above. However, starting from T_2_, modifications were made to the Youth Screen Time Survey to accommodate the increasing DM usage among school-aged children. The time spent watching television was changed into ‘watching or streaming videos or movies’, while watching videos (such as YouTube) was changed into ‘watching or streaming videos or live streaming (such as YouTube, Twitch)’. Then, these categories were merged into a single category named ‘watching television/videos’. ‘Video chatting, visiting social media apps, and texting cell phone’ were combined into a broader category called ‘using social media’. The activities ‘editing photos and videos’ and ‘searching or browsing the internet’ were excluded as they were not present in the T_0_ data. Playing video games was further divided into two subcategories, i.e., ‘time spent on single-player’ and ‘time spent on multi-player’, which were combined as ‘playing video games’.

Additionally, the response format was changed from categorical to continuous, with response options including 0 min, 15 min, 30 min, 45 min, 1 h, 1.5 h, 2 h, 2.5 h, 3 h, and every additional hour up until 24 h.

To ensure consistency across all time points, we standardized the T_2_, T_3_, and T_4_ visit data to align with the T_0_ and T_1_ visit data. As a result, the data from the T_2_, T_3_, and T_4_ visits were recoded to match the categories used in the T_0_ and T_1_ visits. The recoding involved transforming the continuous response options into the following categories: none—‘0’, < 30 min—‘0.25’, 30 min—‘0.5’, 1 h—‘1’, 1.15 h—‘1.25’, 1.30 h—‘1.5’, 2 h—‘2’, 2.15 h—‘2.25’, 2.30 h—‘2.5’, 3 h—‘3’, 3.15 h—‘3.25’, 3.30 h—‘3.5’, and > 4 h—‘4’.

There were good test–retest correlations between individual DM usage across different time points, with r-values ranging from 0.24 to 0.56 (eFigure 1).

### Parent-reported survey

Caregivers/parents were asked to report the number of hours spent by their child on a typical weekday and weekend day engaging in total on watching television, shows or videos, texting or chatting, playing games, or visiting social networking sites (Facebook, Twitter, Instagram), excluding the number of hours spent on school-related work during T_0_ and T_1_ visits. Parents provided the total estimated time spent on these activities in both hours and minutes for weekdays and weekends. To calculate the average hours of screen time per day, we used the following: [(total number of hours spent on a weekday * 5) + (the total number of hours on a weekend day * 2)]/7.

Furthermore, we assessed the agreement between caregivers/parents and child reports regarding the estimated amount of time spent on total screen activities (i.e., watching television/videos, playing video games, or engaging in social media) during the T_0_ visit, using a correlation coefficient and found it to be 0.37, indicating fair agreement between caregivers/parents and children. To obtain the child’s report on the estimated screen time at T_0_, we summed the time spent watching television/videos, playing video games, or using social media.

We opted to use the self-reported surveys completed by the children rather than relying on caregivers/parents^74,75^. Since caregivers/parents may not be fully aware of specific types of DM used by their children, including those aged 9–11 years and older, who often use DM without supervision, such as in their bedrooms at night. Consequently, children may provide more accurate reports of their estimated time spent on each type of DM usage. There is also substantial evidence showing that children as young as 6 years old can reliably report on their own health^75^.

In light of the COVID-19 lockdown, it is probable that these children could spend more time using DM than anticipated at T_0_. This effect was more pronounced in a US-based study, which reported a two-fold increase in the estimated time spent on DM usage during the COVID-19 lockdown compared to the pre-pandemic period^76^. Therefore, to account for an increase in estimated time spent using DM among children between T_0_ and T_4_, we used the average estimated time spent for individual DM usage, rather than relying solely on data from either T_0_ or T_4_ for the longitudinal analyses. The average estimated time spent for individual DM usage was calculated by averaging the estimated time spent for each type of DM usage across all time points.

### Outcomes

These predefined outcomes included the global CSA and the volumes of the striatum and cerebellum. We defined the striatum by combining the volumes of the caudate nucleus, putamen, and accumbens. As for the cerebellum, we combined the volumes of both grey and white matter structures of the cerebellum. Both striatum and cerebellum volumes were adjusted for the total brain volumes. In these analyses, we considered both the left and right hemispheres together.

### Statistical analysis

Descriptive statistics including means and standard deviations (SDs) were calculated.

The first research question aimed to assess whether individual DM usage altered (i.e., increased or decreased) brain development over 4 years.

To address this question, we first inspected the developmental trends of brain structures (i.e., global CSA, cerebellum, and striatum) between mid-childhood and early adolescence, which are not always linear. Earlier studies on brain development have reported both linear and quadratic trends between childhood and adolescence^77–80^. To do so, we compared the default linear model to a complex quadratic model to identify whether adding the quadratic age effect significantly improved the goodness of fit for the global CSA, cerebellum, and striatum. In both these models, we adjusted for SES, polygenic scores cogPGS, and 20PCs. We assessed the fit of the models based on the Akaike Information Criterion (AIC) and Bayesian Information Criterion (BIC). The model with lower AIC and BIC values was considered a better fit (at least by 10 points less than the other model) (eTable 1)^81^. The log-likelihood ratio test (χ^2^) was additionally run to confirm the results.

When we examined the models, the quadratic model fitted the data well and was subsequently used for further analysis. In addition, age-related change in the peak location along with sex effect was assessed. Peak age for each brain structure was calculated using the first derivative of the quadratic equations.

We constructed a quadratic mixed-effect model to investigate the relationship between individual DM usage and brain structures over time. The model (Eq. 1) was adjusted for various factors: age at baseline (mean-centered to reduce multicollinearity), SES, cogPGS, 20 PCs, and sex assigned at birth as fixed effects, and study sites were included as random effects.

To test the long-term effect of DM usage on brain development with time (as outcomes of interest), we included a two-way interaction with average DM usage and time as both linear and quadratic terms (i.e., average DM usage x Time; average DM usage x Time^2^). Furthermore, to account for SES and cogPGS effects on brain development over time, we included three-way interactions in the same model (i.e., for SES, average DM usage x Time x SES; and average DM usage x Time^2^ x SES; for cogPGS, average DM usage x Time x cogPGS; and average DM usage x Time^2^ x cogPGS). Both the intercepts and the slopes were used as random-effects terms, allowing children to start at different levels of surface area/volumes. The ‘lmer’ function of package lme4 in R software was used to fit the model, and the restricted maximum likelihood method was used to estimate the model parameters^82,83^.

1 $${y}_{ij}={\beta }_{0}+{\beta }_{1}{cogPGS}_{i}+{\beta }_{2}{t}_{ij}+{\beta }_{3}{Sex}_{i}+{\beta }_{4}{Age\, at\, baseline}_{i}+{\beta }_{5}{DM\, Usage}_{i}+ {\beta }_{6}{SES}_{i}+ {\beta }_{7}{Ancestry(20PCs)}_{i}+ {\beta }_{8}\left({DM\, Usage}_{i} * {t}_{ij}\right)+{\beta }_{9}\left({DM\, Usage}_{i}*{t}_{ij}*{cogPGS}_{i}\right)+{\beta }_{10}({DM\, Usage}_{i}*{t}_{ij}*{SES}_{i}) {+ \upsilon }_{0i}+{ \upsilon }_{1i }* {t}_{ij}+{\varepsilon }_{ij}$$

*β*_0_ represents the intercept; *β* represents the parameter estimate, ${cogPGS}_{i}$ represents the polygenic scores for cognitive performance of a child *i;* ${t}_{ij}$ represents the effect of time (denotes the follow-up time for child *i* at visit *j*, fitted as a continuous measure in years; ${Sex}_{i}$ sex of a child, dummy coded *(1* = *M, 0* = *F);* ${Age}_{i}$ age of child *i as a continuous measure* at baseline; ${SES}_{i}$ represents the socioeconomic status for child *i;* ${Ancestry}_{i}$ represents the ancestry differences in genetic structure that could bias the findings; ${DM Usage}_{i}$ represents the amount of average estimated time spent for child *i,* fitted as a continuous measure; ${\upsilon }_{0i}$ and ${\upsilon }_{1i}$ are the random effects, and ${\varepsilon }_{ij}$ is the random error term at the *j*th time point for child *i.*

To determine the effect of sex-related differences on the relationships between DM usage and brain development, we added an interaction effect of sex (i.e., average DM usage x Time x sex; and average DM usage x Time^2^ x sex) to the pre-existing model (Eq. 2).

2 $${y}_{ij}={\beta }_{0}+{\beta }_{1}{cogPGS}_{i}+{\beta }_{2}{t}_{ij}+{\beta }_{3}{Sex}_{i}+{\beta }_{4}{Age\, at\, baseline}_{i}+{\beta }_{5}{DM\, Usage}_{i}+ {\beta }_{6}{SES}_{i}+ {\beta }_{7}{Ancestry(20PCs)}_{i}+ {\beta }_{8}\left({DM\, Usage}_{i} * {t}_{ij}\right)+{\beta }_{9}\left({DM\, Usage}_{i}*{t}_{ij}*{cogPGS}_{i}\right)+{\beta }_{10}({DM\, Usage}_{i}*{t}_{ij}*{SES}_{i}) {+ {\beta }_{10}({DM Usage}_{i}*{t}_{ij}*{Sex}_{i})+ \upsilon }_{0i}+{ \upsilon }_{1i }* {t}_{ij}+{\varepsilon }_{ij}$$

Considering the numerous statistical tests conducted, the Bonferroni corrections were applied to control for Type-1 error^84^. In total, we performed three individual DM usage models (i.e., using social media, playing video games, watching television/videos) for three brain structures analyzed in the overall cohort as well as for sex, resulting in a total of 18 tests. *P* < 0.003 was considered statistically significant.

An additional analysis was conducted to investigate whether the estimates of DM preceded changes in cerebellum volume. A linear model was employed to ascertain whether the average social media usage of the first two time points (i.e., (T_0_ + T_1_)/2) could predict later changes in cerebellum volumes between T_2_ and T_4_, while adjusting for the aforementioned covariates (i.e., age at baseline, SES, cogPGS, 20 PCs, and sex assigned at birth as fixed effects, and study sites as random effects). Subsequently, the same analysis was repeated using the average time spent playing video games during the first two time points in cerebellum volumes. We then investigated whether social media usage at T_0_ could predict the changes in cerebellum volumes (T_4_–T_0_) over the study period, while adjusting for prespecified covariates as mentioned above. In addition, we explored whether excluding time spent on video chatting or texting from social media usage would alter the results (Eq. 1; Table 2).

We ran multiple robustness tests to validate our findings and they were uncorrected. Firstly, we excluded children who were born preterm (< 37 weeks), had low birth weight (< 2500 g) or had a diagnosis of ADHD. Those born preterm or with low birth weight tend to have altered developmental trajectories^85–87^. Similarly, children with ADHD have delayed maturation, which might affect our findings^88^. Secondly, we restricted our analysis by including children with MRI data for all three-time points.

The gestation length and birth weight of each child were reported by caregivers/parents through a self-reported questionnaire. The presence of ADHD symptoms in the child, whether in the past or currently, was assessed through caregivers/parents reports using the computerized Kiddie-Structured Assessment for Affective Disorders and Schizophrenia (KSADS) during the T_0_ visit. This tool is based on a well-studied and validated tool, both in research and clinical settings. Diagnoses of ADHD were made in accordance with *DSM-5* criteria, which require an endorsement of six or more symptoms of inattention or hyperactivity-impulsivity.

### Supplementary Information

Supplementary Information.

## Data Availability

The data used for the analyses presented in this paper are from the Adolescent Brain Cognitive Development (ABCD) Study [https://abcdstudy.org; NIMH Data Archive (NDA)]. Data can be accessed by directly applying to the NDA.

## Code availability

The code to replicate all analysis described in this manuscript can be found here: https://github.com/samniv/ScreenTime-and-Brain/tree/main.
